# Emergence of new antigenic epitopes in the glycoproteins of human respiratory syncytial virus collected from a US surveillance study, 2015–17

**DOI:** 10.1038/s41598-019-40387-y

**Published:** 2019-03-07

**Authors:** Bin Lu, Hui Liu, David E. Tabor, Andrey Tovchigrechko, Yanping Qi, Alexey Ruzin, Mark T. Esser, Hong Jin

**Affiliations:** 1 MedImmune/AstraZeneca, South San Francisco, CA USA; 2 MedImmune/AstraZeneca, Gaithersburg, MD USA

## Abstract

Respiratory syncytial virus (RSV) is a significant cause of lower respiratory tract infection in infants and elderly. To understand the evolution of neutralizing epitopes on the RSV glycoprotein (G) and fusion (F) proteins, we conducted a multi-year surveillance program (OUTSMART-RSV) in the US. Analysis of 1,146 RSV samples from 2015–2017 revealed a slight shift in prevalence from RSV A (58.7%) to B (53.7%) between the two seasons. RSV B was more prevalent in elderly (52.9% and 73.4%). Approximately 1% of the samples contained both RSV A and B viruses. All RSV A isolates were ON1 and almost all the B isolates were BA9 genotypes. Compared with the 2013 reference sequences, changes at the F antigenic sites of RSV B were greater than RSV A, which mainly occurred at antigenic sites V (L172Q/S173L at 99.6%), Ø (I206M/Q209K at 18.6%) and IV (E463D at 7%) of RSV B F. Sequence diversities in the G protein second hypervariable region were observed in the duplicated regions for RSV A and B, and at the G stop codon resulting in extension of 7 amino acids (22.1%) for RSV B in 2016–17. Thus, RSV surface glycoproteins are continuously evolving, and continued surveillance is important for the clinical evaluation of immunoprophylactic products.

## Introduction

RSV is the most common cause of lower respiratory tract infections (LRTI) among infants and young children worldwide, resulting in annual epidemics worldwide^1^. In the United States (US) alone, there are estimated 132,000 to 172,000 RSV-associated hospitalizations annually involving children <5years-old, predominantly in infants^2^. The only approved anti-RSV immunoprophylaxis is Palivizumab^3,4^, a humanized monoclonal antibody (mAb) directed against the RSV F protein, which is licensed only for infants at highest risk. Although Palivizumab has been shown to reduce hospitalization by 50% in high-risk infants^5^, prevention of RSV illnesses in all infants remains a major public health priority. RSV is also an important causative agent of LRTI in the elderly. In the US, among the annual 687,000 hospitalizations and 74,000 deaths caused by pneumonia in the elderly, approximately 2–9% of these are caused by RSV^6,7^.

RSV belongs to the Paramyxoviridae family. It is a non-segmented, negative-sense, single-stranded, enveloped RNA virus. RSV virions contain three glycoproteins, F, G and SH. The F and G proteins are the two major integral membrane proteins that play critical roles in viral entry and egress, and are the major antigens for eliciting neutralizing antibody responses. Although the G protein is dispensable for RSV replication *in vitro*, G deletion mutants are highly attenuated *in vivo*^8^, suggesting a critical role of the G protein in infecting human airway epithelial cells. RSV is classified into two subtypes, RSV A and RSV B, based on the genetic characteristics of the G gene^9^. The C-terminal region of the G protein (the second hypervariable region, HVR2) has been used as the basis for genotyping^10^. RSV A ON1 genotype, characterized by a 72 nucleotide (nt) (23 amino acid residues, AA) duplication in the HVR2, is currently the predominant RSV A genotype. It was first reported in Canada in 2012^11^ and had been detected in at least 25 countries by 2017^12–15^. RSV B BA genotype, characterized by a 60 nt (20 AA) duplication in the HVR2, was first reported in Argentina in 1999 and has become the predominant RSV B genotype globally^16^. The emergence of these two distinct RSV subtypes independently has been hypothesized to be due to immune pressure on the G protein selecting for these novel viruses^17–19^.

The RSV F protein is essential for viral entry into cells as it mediates fusion between virion and host cell membranes. Fusion requires a conformational change from the pre-fusion to the post-fusion form of F^20^. Targeting the transition from the pre-fusion F to the post-fusion F form can prevent the fusion process and block RSV infection *in vitro* and *in vivo*^18,21–23^. Several mAbs and small molecules targeting the pre-fusion form of F are currently under clinical development and could become effective prophylactic or therapeutic interventions against RSV^18,21–23^. The F protein contains several antigenic sites (Ø, I-V)^24,25^. Two antigenic sites, II (target of Palivizumab) and IV (target of 101F and MAb19), are present in both the pre- and post-fusion F proteins^26,27^. However, the pre-fusion and post-fusion F proteins also harbor their own respective antigenic sites such as site Ø (target of MEDI8897^18^) in pre-fusion form of F and site I in post-fusion form of F, respectively^28^. Monoclonal antibodies such as Motavizumab against site II^29,30^ and Suptavumab against site V^31,32^ have also been tested clinically. Although the F protein is generally thought to be conserved, variability in the F protein leading to viruses that can escape neutralization by anti-RSV mAbs has been described^30,33–36^.

To understand the RSV molecular epidemiology and the potential impact of genetic variability on vaccine and antiviral drug development, we collected and sequenced RSV-positive samples from the US and Puerto Rico as part of the OUTSMART-RSV (Observational United States Targeted Surveillance of Monoclonal Antibody Resistance and Testing of Respiratory Syncytial Virus) surveillance program.

## Results

### Differential distribution of RSV A and RSV B subtypes

A total of 1,146 RSV positive samples from subjects ranging in age from birth to 97 years were subtyped and revealed that both RSV A and B co-circulated in both the 2015–16 and 2016–17 winter RSV seasons. RSV A (58.7%) was more prevalent than RSV B (40.0%) in the 2015–16 season whereas RSV B (53.7%) was more prevalent than RSV A (45.3%) in the 2016–17 season. In both seasons, a small number of samples contained both RSV A and B [n = 4 (1.3%) in 2015–16 and n = 8 (1.0%) in 2016–17] (Table 1).

**Table 1:** Demographic characteristics of patients with RSV A, RSV B and A/B co-infections from the 2015–16 and 2016–17 RSV seasons in the US.

	2015–16 RSV Season				2016–17 RSV Season			
	Total	RSV A	RSV B	RSV A/B	Total	RSV A	RSV B	RSV A/B
	N	N (%)	N (%)	N (%)	N	N (%)	N (%)	N (%)
N (%)	310	182 (58.7%)	124 (40.0%)	4 (1.3%)	836	379 (45.3%)	449 (53.7%)	8 (1.0%)
**Age Group**								
<1 year	173	105 (60.7%)	67 (38.7%)	1 (0.6%)	516	237 (45.9%)	273 (52.9%)	6 (1.2%)
1–2 year	45	28 (62.2%)	17 (37.8%)	0 (0.0%)	150	88 (58.7%)	62 (41.3%)	0 (0.0%)
3–59 year	75	42 (56.0%)	31 (41.3%)	2 (2.7%)	106	37 (34.9%)	67 (63.2%)	2 (1.9%)
>60 year	17	7 (41.2%)	9 (52.9%)	1 (5.9%)	64	17 (26.6%)	47 (73.4%)	0 (0.0%)
**Gender**								
male	176	107 (60.8%)	67 (38.1%)	2 (1.1%)	442	202 (45.7%)	234 (52.9%)	6 (1.4%)
female	134	75 (56.0%)	57 (42.5%)	2 (1.5%)	394	177 (44.9%)	215 (54.6%)	2 (0.5%)

The majority (60.1%) of the samples were from infants <1 year-old with the remainder of samples from subjects between 1 and 97 years. There were more male than female patients in both RSV seasons overall. Noticeably, during the 2015–16 season when RSV A was more prevalent, RSV A was detected in more younger patients, 60.7% in <1 year-old, 62.2% in 1 to 2 years-old, 56.0% in 3 to 59 years-old, but lower in >60 years-old (41.2%). In contrast, during the 2016–17 season when RSV B was more prevalent, RSV A was still most prevalent in the age group 1 to 2 years-old (58.7%). The percentage of RSV B positive samples in other age groups were 52.9% in <1 year-old, 63.2% in 3 to 59 years-old and 73.4% > 60 years-old, respectively (Table 1). Interestingly, RSV B in subjects >60 years-old was higher than RSV A in both seasons (52.9%, in 2015–16; 73.4%, in 2016–17), suggesting preferential RSV B infection in older adults.

### Phylogenetic analyses of RSV A and B fusion proteins

**Figure 1:**
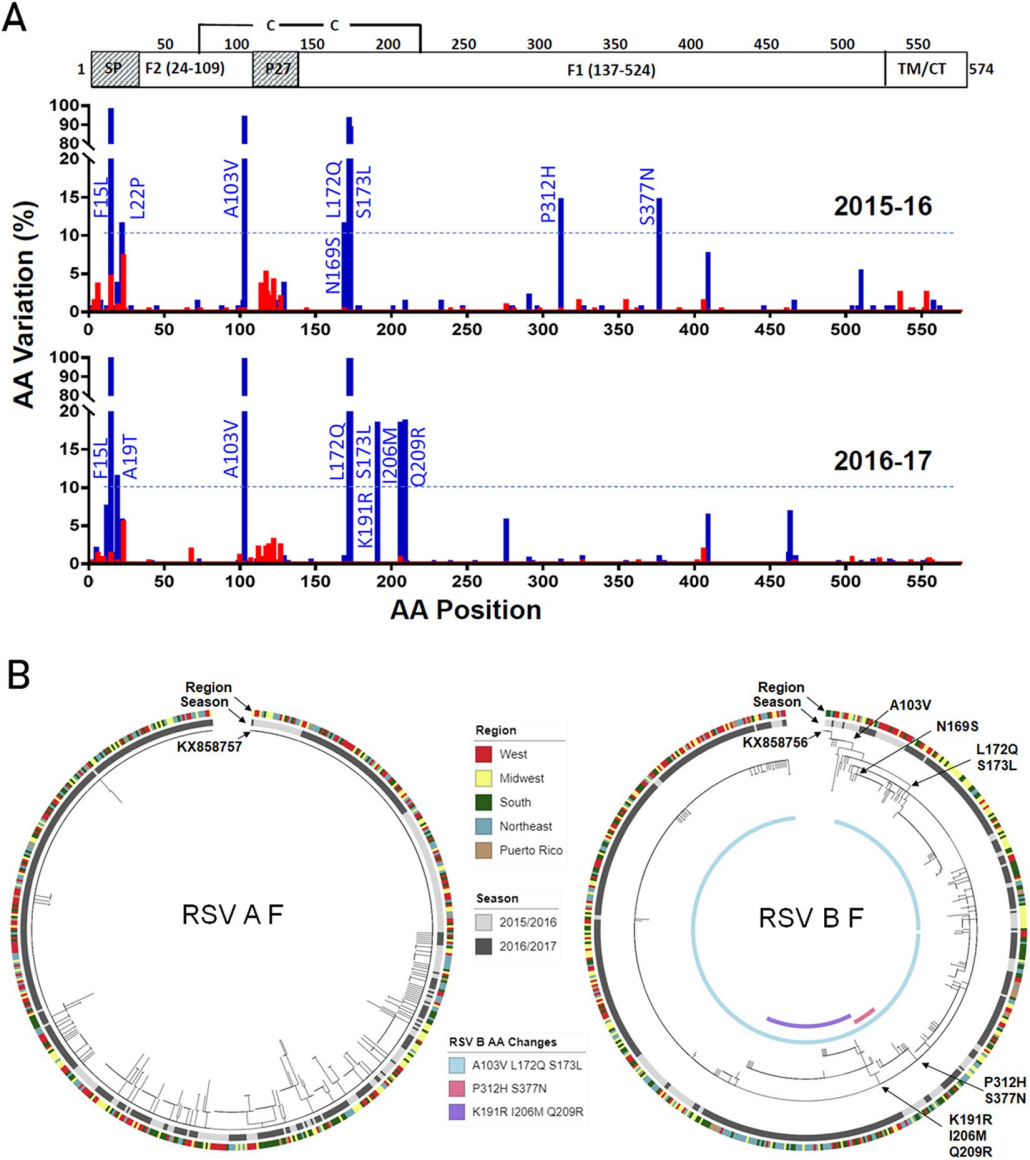
Polymorphisms in the RSV F protein. (**A**) Plots of AA variation frequency by position of F protein ORF (1–574) to the KX858757.1 and KX858756.1 reference sequences. The functional domain structures illustrated on the top (SP, signal peptide; TM, transmembrane domain, CT, cytoplasmic tail). OUTSMART-RSV samples from 2015–16 and 2016–17 are plotted separately. Red, RSV A; Blue, RSV B. Variations at a frequency >10% (dash lines) in each season relative to the references are labelled. (**B**) Phylogenetic trees of RSV A F and RSV B F proteins. Reference sequences KX858757.1 and KX858756.1 for RSV A and RSV B, repectively, are used to root the trees. Metadata of each samples including years of isolation and their geographic location are annotated. AA substitutions for the major branches are labeled.

A total of 1,158F protein sequences (561 RSV A, 573 RSV B and 12 RSV A/B co-infections) from the two RSV seasons were aligned against the reference sequences derived from two 2013 clinical isolates RSVA/13–005275 (GenBank accession no. KX858757) and RSVB/13–001273 (GenBank accession no. KX858756). AA variation and phylogenetic analysis of the F protein sequences for RSV A F and RSV B F are summarized in Fig. 1. Overall, there was more genetic diversity in the RSV B F than the RSV A F. Out of the 574 AA residues in the entire F open reading frame (ORF), RSV A F showed variations in 45 (7.8% in 2015–16) and 63 (11.0% in 2016–17) positions in different viruses (ranging from 1–4 changes in any single virus compared with the reference strain). On the other hand, RSV B F had 56 (9.8% in 2015–16) and 91 (15.9% in 2016–17) amino acid positions in different viruses (ranging from 1–11 changes in any single virus relative to the reference strain) during these two seasons, respectively (Fig. 1A).

Since the extracellular region of the mature F protein (AA 24–109, 137–524) is the target of multiple anti-RSV mAb and vaccines in clinical development^24,37^, we compared the OUTSMART-RSV samples with the reference sequences for the extracellular region. Specifically, 161 out of the 186 RSV A F (86.6%) of the 2015–16 OUTSMART-RSV samples and 322/387 (83.2%) of the 2016–17 samples were identical to the mature F protein reference strain A/13-005275. Sequence diversity was noted in the signal peptide (AA 1–23) and cleaved P27 (AA 110–136) but none of the AA changes in the entire ORF had frequencies greater than 10% among the RSV A viruses compared with the 2013 RSV A reference strain (Fig. 1A). Phylogenetic analysis of the RSV A samples from both RSV seasons relative to their geography indicated that these variations in RSV A were neither temporally nor geographically distinct (Fig. 1B).

In contrast, RSV B F sequences from 2015–16 strains showed that the majority (88.3%, 113/128) contained A103V, L172Q and S173L changes at site V compared with the 2013 RSV B reference (Fig. 1A). These three changes likely emerged in the 2014–2015 RSV season as they are not seen in RSV F sequences before 2013 in the NCBI database (data not shown). Almost all samples (99.6%, 455/457) from 2016–17 had these three substitutions. Other AA changes in the RSV B F with a frequency greater than 10% included N169S (11.5%), P312H (14.5%) and S377N (14.5%) in the mature protein of RSV samples collected from the first season. Unlike the A103V/L172Q/S173L changes that were present in RSV B samples in the second RSV season, P312H and S377N had decreased from 14.5% in the first season to 0% and ~1% in the second season, while K191R (18.6%), I206M (18.6%) and Q209R (18.6%) appeared at a frequency greater than 10% (Fig. 1A).

Phylogenetic analysis of the RSV B F proteins showed that several AA changes occurred together. In addition to the A103V/L172Q/S173L changes present in almost all the F sequences, N169S, P312H/S377N and K191R/I206M/Q209R formed distinct clades (Fig. 1B). The samples containing P312H/S377N were almost all from the 2015–16 season (pink) and K191R/I206M/Q209R were exclusively from the 2016–17 season (purple). There was no clear correlation of the AA substitutions with geographic locations, suggesting that these different strains were not confined in specific regions in the US (Fig. 1B).

### Emergence of novel F protein epitopes in site Ø, I – V

**Figure 2:**
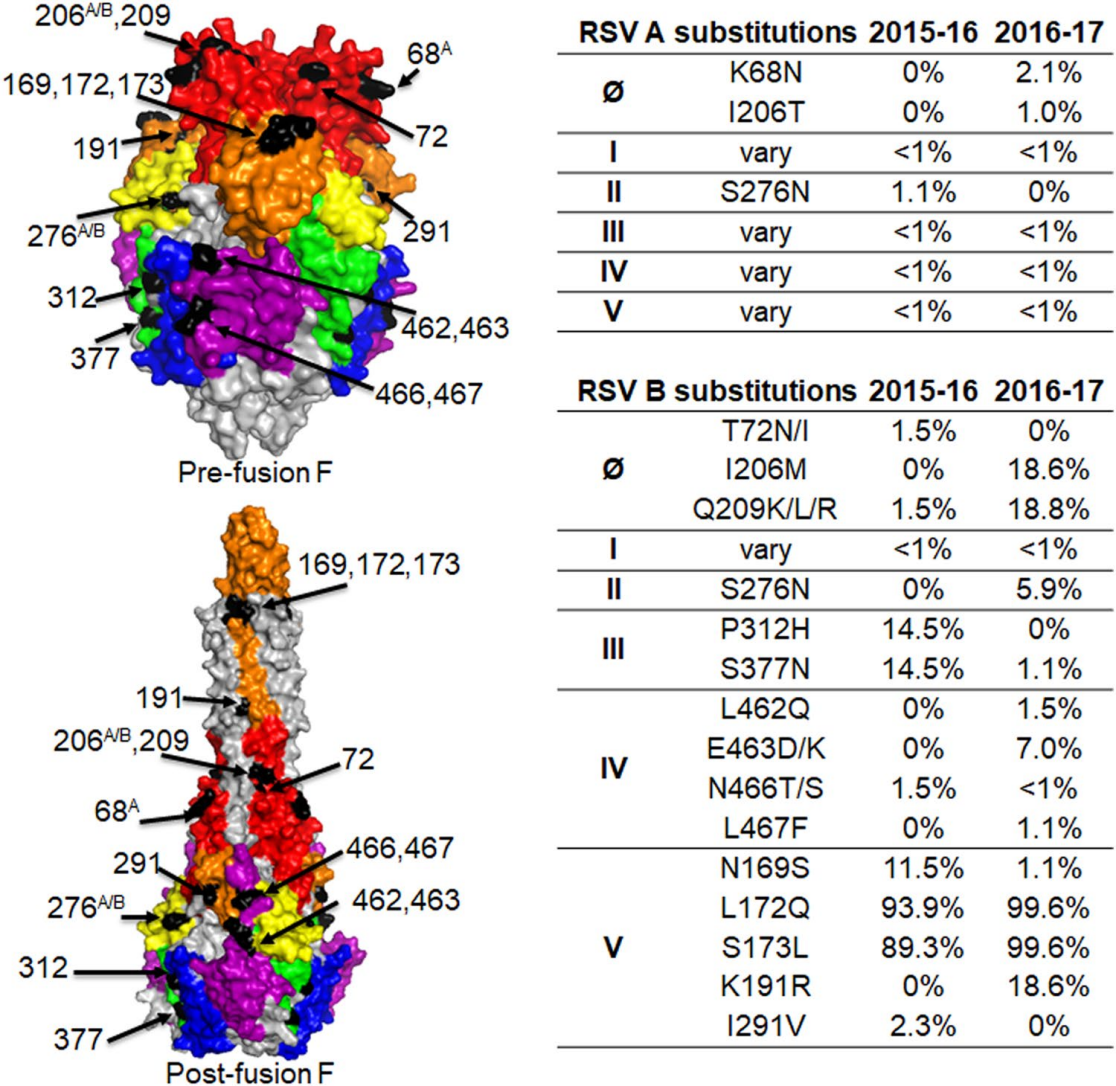
Polymorphisms in antigenic sites of RSV F proteins. RSV F protein pre-fusion and post-fusion conformations were based on the pdb files of 5UDE and 3RRR, respectively. Antigenic sites (Ø, I, II, III, IV and V) are surface-color-coded. red: site Ø, blue: I, yellow: II, green: III, purple: IV, orange: V. Amino acid variations in these antigenic sites and their frequency for each season are listed in the tables on the right. Only sites with variations >1.0% are labeled on the 3D structures^37^. (**A**): indicates variation only present in RSV A. (**A**,**B**): indicates residues that showed variations in both RSV A and RSV B. Other numbers indicate variations in RSV B only.

The RSV F protein contains six major antigenic sites (Ø, I-V)^24,25,38^. AA variations at these sites with frequency >1% in their respective seasons of the OUTSMART samples are labelled on the F protein structures for both the pre-fusion and post-fusion forms (Fig. 2, left panels). There were 10 and 16 amino acid variations across the antigenic sites of RSV A F protein over the two RSV seasons, respectively. Changes present at a frequency >1% included the K68N and I206T in site Ø and S276N at site II of RSV A F. In contrast, there were 19 and 34 different amino acid changes in the antigenic sites of RSV B F proteins for these two seasons, 15 of which had frequencies >1% at different antigenic sites (Fig. 2). These include double I206M/Q209R and single Q209K or Q209L substitutions at site Ø, P312H, S377N at site I, S276N at site II, L462Q, E463D/K, N466T/S, L467F at site IV, and S169S, L172Q, S173L, K191R and I291V changes at site V. AA at positions 169, 172, and 173 are in close proximity to one another in the pre-fusion F structure; AA 191 is distal to the other 3 AA in the post-fusion structure and closer to site Ø (Fig. 2, left panel). The I206M/Q209R at site Ø and K191R at site V emerged together during the second OUTSMART-RSV season. L172Q/S173L at the antigenic site V was observed in most of the 2015–16 samples (89.3%) and increased to 99.6% in the following 2016–17 season. The L172Q/S173L double substitutions were not present in RSV F sequences prior to 2014 in the NCBI database (data not shown). Several novel viruses appeared in 2016–17 including the strains carrying the I206M/Q209R double substitutions at site Ø along with a triple AA change (K191R/ L172Q/S173L) at site V. Interestingly, P312H and S377N at site III appeared in 2015–16 at 14.5% but disappeared in the following season. In summary, these data show that the RSV F protein is generally conserved, but there is evidence for continued changes at key antigenic sites on the F protein.

### Phylogenetic analyses of the G protein second hyper-variable region of RSV A and B

**Figure 3:**
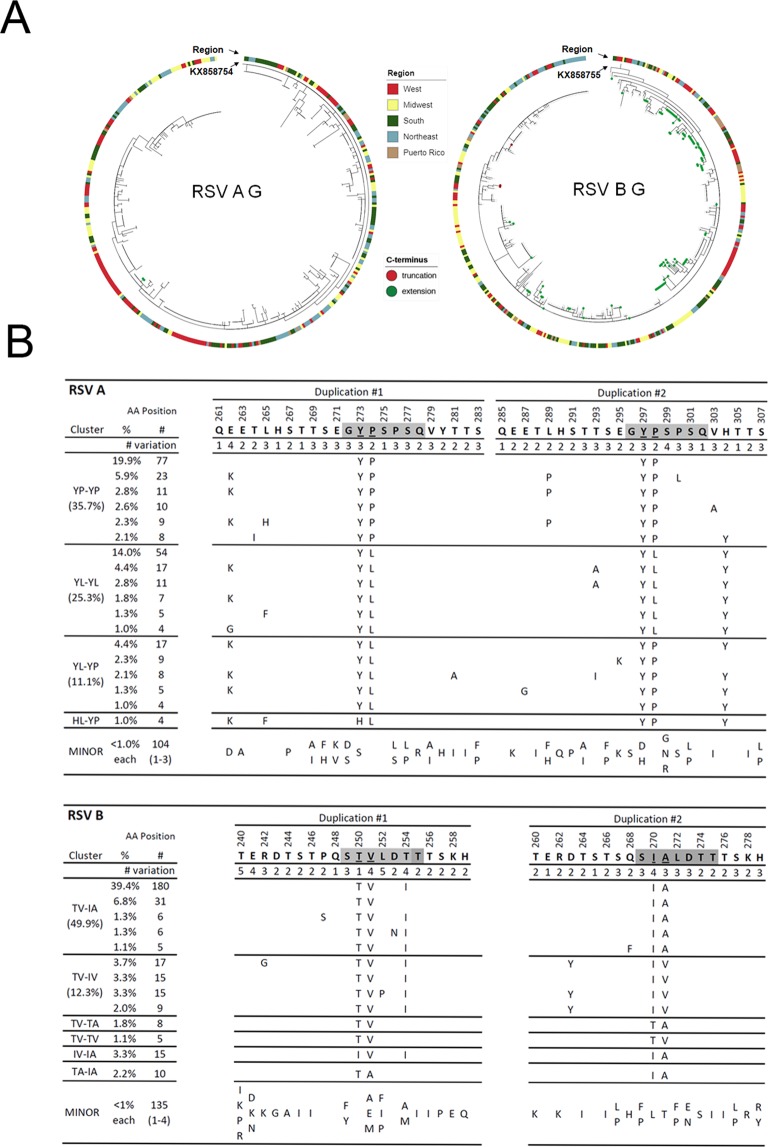
Sequence variations of the second hypervariable region of G protein [G-HVR2] and motif clusters of the duplications in G-HVR2. (**A**) Phylogenetic trees based on RSV A G and RSV B G HVR2 sequences. Reference sequences KX858754.1 and KX858755.1 for RSV A and B, respectively are used to root the trees. Geographic metadata of the samples are depicted on the outer-rim of the trees. Nodes with green dots indicate G proteins with extra amino acid extensions. Nodes with red dots indicate G proteins with truncations due to pre-mature stop codons in the ORF. (**B**) Pairwise sequence alignment of the duplicated regions in the G HRV2 of RSV A and RSV B. The duplication regions (AA 261–283 and 285–307 for RSV A; AA 240–259 and 260–279 for RSV B are indicated as duplication #1 and duplication #2. Motifs used for the analysis are highlighted with a grey shade. Clustering was performed based upon the sequences in the duplicated regions. Blank spaces indicate identical amino acids with the chosen reference for RSV A and RSV B, respectively. Percentages of each representative cluster for RSV A and RSV B are indicated.

Phylogenetic analysis of 836 samples from the 2016–17 RSV season using reference sequences of known G genotypes revealed that all RSV subtype A isolates were ON1 genotype and almost all RSV B isolates were of genotype BA9 except for one BA10 isolate (Fig. 3A). These findings are consistent with previously published reports that RSV ON1 and RSV BA9 genotypes have emerged as the predominant strains in the US and throughout the world^12–15^. Annotating the phylogenetic trees with the regions of the clinical samples collected showed that some sequences clustered together by geography. For example, some samples from the West (red) are clustered in clades in the left lower corner of the RSV A G tree. Overall, there is no clear geographic distribution differences among all the isolates.

Sequence analysis revealed multiple forms of the G protein with different lengths that were more frequent in RSV B than RSV A isolates (Fig. 3A). For RSV A, a single nucleotide mutation in the stop codon TGA to TTA (L) was observed in 2 samples (2/387, 0.5%) resulting in an extension of the G protein by 1 AA. In contrast, 101 of 457 (22.1%) RSV B samples had a mutation in the stop codon TAA to CAA (Q) resulting in the G protein extension by 7 AA (Q/K)-R-L-Q-S-Y-(H/A) (green dots in Fig. 3A). In addition, three RSV B samples (3/457, 0.7%) had a premature stop codon (GAG- > TAG) that shortened the G protein by 7 AA (deleting “P-S-T-S-N-S-T”, red dots in Fig. 3A).

All RSV A and B samples had the characteristic ON1 and BA genotype duplications in the G HVR2 with either 23 AA (AA 261–283 and 285–307 for RSV A)^11^ or 20 AA (AA 240–259 and 260–279 for RSV B)^38^. These duplicated regions have several highly conserved sequence motifs that can be used as markers to evaluate genetic diversity and viral evolution. One of the conserved motifs, “G**YP**SPSQ” of RSV A G in the first duplicated region, is identical to the corresponding sequence of the second duplicated region of the 2013 reference strain (Fig. 3B). Although 37.3% isolates had the same signature motif defined as “YP-YP” in both duplicated regions, other motif variations including “YL-YL” (25.3%), “YL-YP” (11.1%) and “HL-YP” (1.0%) were observed, suggesting continued evolution in this key neutralizing region of the G protein.

Similarly, the G protein of the RSV B reference strain contained conserved sequences in the duplicated regions, “S**TV**LDTT” in the first duplicated region and “S**IA**LDTT” in the second duplicated region (Fig. 3B). The majority of RSV B isolates (49.9%) contained the “TV-IA” sequence; “TV-IV” (12.3%) is the next major cluster, only 1.1% samples contained the same “TV-TV” sequence. Other combinations and mixed residues were also observed, suggesting constant evolutionary changes in the G gene. There are no clear geographic distribution differences among all the sequences. These data suggest that immune pressure on the G protein has led to continued evolution of the second hypervariable region with multiple strains now in circulation.

## Discussion

The evolution of RSV strains is a dynamic process that involves periodic alternation in the dominance of subtypes A and B as well as sequence diversity in the G attachment protein^10^. In the OUTSMART-RSV study we showed that RSV A infection was more prevalent in subjects younger than 60 years old (<1, 1–2 and 3–59 years) in the 2015–16 season compared with the 2016–17 season (Table 1). However, RSV B was more prevalent in subjects greater than >60 years old in both seasons (52.9% in 2016–2017 and 73.4% in 2016–17). The difference in prevalence of RSV B in 1 to 2 year-old (62/150, 41.3%) versus >60 year-old group (47/64, 73.4%) was statistically significant (p < 0.0001). This data suggest that pre-existing immunity to RSV B in older people may be poorer than RSV A or due to the lower immunity to antigenically drifed comptaporary RSV B strains in the elderly^39^. Although the sample size of the older adult group was smaller than the infant group and 1 to 2 year-old group, sequence analysis of 81 isolates collected from the elderly provides a valuable set of data for comparison with the future isolates as we will continue characterizing RSV A and B isolates from different age groups in on-going global surveillance studies.

Immune pressure and lack of proof-reading capabilities of the viral RNA-dependent polymerase of RSV likely drive RSV evolution. This evolution is reflected in the sequence diversities in key antigenic regions of the F and G proteins. RSV genotypes have gone through many dynamic changes over the past several decades, wherein newly emergent genotypes replaces the older ones^40,41^. Both RSV A ON1 and RSV B BA genotypes represented by the duplicated sequence in the C-terminal G protein emerged independently at different times and became predominant RSV genotypes globally^16,42^. These genotypes have been suggested to alter viral antigenic properties to facilitate viral infection by evading the host immune response^43^, to enable virus spread efficiently or conferring a fitness advantage during circulation^12^.

The fast spread of the RSV A ON1 and RSV B BA genotypes has been accompanied with diversifications of the G protein within the genotypes such as protein length variation and sequence diversity in the duplication regions^44,45^. The biological and immunological significance of these extra amino acids in the G protein extracellular domain remains to be determined. The first reported RSV ON1 genotype (ON138–0111A) in 2012 had an identical 23-AA duplication (261–283 and 285–307)^11^. Similarly, the first reported RSV BA genotype (BA3833/99B) isolated in 1999 had an identical 20-AA duplication (240–259 and 260–279)^38^. The divergent sequences observed in these two duplicated regions of the G protein have been hypothesized to either occur in a convergent fashion^40^ or via multiple independent duplication events.

Although the F protein of RSV is more conserved compared with the G protein, variations at key antigenic sites have been identified in the F proteins of RSV A and B samples in this study (Fig. 2). Noticeably, the RSV B containing A103V/L172Q/S173L in the F protein emerged around 2014 has become the predominant circulating RSV B strain in the US. These substitutions were also reported in the RSV B samples during a similar surveillance period in China^46^, suggesting global circulation of this new RSV strain. Antigenic site Ø is the binding site for an mAb (MEDI8897) targeting the prefusion form F^18,25,47^ that is currently being evaluated in infants in Phase II clinical study. For the first time, we identified the emergence of the RSV B I206M/Q209R double changes at a frequency of 18.6%. The I206M/Q209R change at site Ø is also accompanied with a K191R change at site V in addition to the A103V/L172Q/S173L changes, suggesting a potential structural or functional relationship of these amino acids. The I206M/Q209R variant has been shown to be susceptible to MEDI8897 neutralization^35^. The other three RSV A and four RSV B variants with distinct polymorphisms in the MEDI8897 mAb binding region identified in this study were at low frequency (<2%) and did not affect viral susceptibility to MEDI8897^35^. Various changes at antigenic site IV at 1–7% frequency and site I at <1% were also identified. These data indicate that none of the antigenic sites are invariant despite high sequence conservation of the F protein.

It is unclear from a structural prospective why some of the changes at the antigenic sites involved multiple residues, such as L172Q/S173L at site V and the A103V change outside of the described antigenic sites. Site V is an attractive target for a mAb and these changes could alter viral antigenicity and affect their susceptibility to mAb targeting this region^48^. Some of the changes occurred in both RSV A and RSV B at the same positions, such as S276N at site II, S377N at site III, which could be due to natural antibodies targeting both A and B subtypes. Other changes appear to come and go, such as S276N in RSV B that increased from 0.8% to 5.9% from the first season to the second season, while S377N in RSV B decreased from 14.5% to 1.1%, and N169S decreased from 11.5% to 1.1%. This data indicates that the 276 and 377 positions might be immunodominant sites for both RSV A and B strains. The changes at F protein antigenic sites may allow viruses to escape neutralization by anti-RSV antibodies from prior RSV infection, which can be examined by testing human sera against the viruses with and without an antigenic change.

The monitoring of the F and G protein antigenic site changes of RSV A and B strains also has implications on RSV vaccine development. In the past, RSV vaccine development has been mainly based on RSV A2 strain, which is a GA1 genotype^49–52^. Given the antigenic site change in the F proteins of RSV B isolates sometimes occurring with a frequency of 100% and the diversity of the G protein, it raises the question if the A2 strain or its F protein alone is appropriate or sufficient to be developed as a vaccine. In addition, there are a number of RSV F-based subunit vaccines in development that use either the pre-fusion or post-fusion forms of the F protein^24^. Characterizing the location of these naturally occurring polymorphisms in sites Ø - V may provide insights into the best antigen(s) to use in an F-based vaccine to protect against both contemporary RSV A and B subtypes.

In conclusion, the OUTSMART-RSV and international surveillance studies enable close monitoring of the molecular evolution of RSV. Characterizing antigenic changes in the RSV F and G proteins and shifts in susceptibility to anti-RSV therapy, will provide important information to help facilitate the development of prophylactic vaccines or therapeutic interventions.

## Materials and Methods

### RSV Sample Collection

The OUTSMART study collected RSV-positive samples from 25 RSVAlert^®^ laboratories from 4 US census regions and Puerto Rico during the winter seasons of 2015–2017. Fourteen (2015–16, season 1) and 25 (2016–17, season 2) regional laboratories in the US and Puerto Rico provided 525 and 1041 RSV positive samples, respectively. A total of 1,146 samples (310 from season 1 and 836 from season 2) were included in the sequencing analysis. All nasal samples were placed in either viral transport medium (VTM) or universal transport medium (UTM) and stored at −70°C. They were shipped frozen on dry ice to a central biorepository, Fisher BioServices, Inc. (Rockville, MD) before being transferred to MedImmune (Mountain View, CA) for sequencing and data analysis.

The nasal sample collection was coordinated by IQVIA who has agreements with the sites participating in OUTSMART and are part of RSVAlert network (https://rsvalert.com). The samples are “diagnostic retains” that are completely de-identified and cannot be linked to the source individuals. Written informed consent was not obtained because sampling was non-invasive. The use of the materials for RSV sequencing was approved by AstraZeneca’s Biological Sample governance committee. All human samples including methods were performed in accordance with the relevant guidelines and regulations.

### Sequencing of RSV G and F Genes

For the 2015–16 samples, RNA was extracted directly from 200 µL of RSV positive nasal samples using viral RNA Mini Kit (Qiagen, Valencia, CA). The RNA was eluted in 60 µL of RNase-free water, and then subjected to RT-PCR amplification of the entire F gene and G HVR2 in separated amplicons (primer sequence and RT-PCR conditions available upon request). Sanger sequencing and contig assemblies were performed at Sequetech Inc. (Mountain View, CA).

For the 2016–17 samples, viral RNA was extracted from 400 μL aliquots of RSV positive nasal specimens and eluted in 100 µL using the Nuclisens easyMAG^®^ instruments (bioMerieux, Durham, NC). The RSV G HVR2 and the F genes were amplified using the purified RNA using the SuperScript III One-Step RT-PCR System (Invitrogen, CA) with forward primer, RSV_F5109–5129Y (5′ AGTGTTCAAYTTYGTWCCYTG 3′) and reverse primer RSV_R7654–7634 (5′ YTACCATTCAAGCAATGACCTC 3′), which were designed to amplify the RSV A and B genomes harboring the G HVR2 and the full-length F gene in a single 2.5 kb amplicon. 10 μL aliquots of amplified RT-PCR product were purified and normalized with the SequalPrep™ Normalization Plate (96) Kit (Invitrogen, CA) and eluted with 20 μL of elution buffer. Approximately 5 μL (~1 ng) of normalized PCR product was used for Nextera Library preparation according to the manufacture’s protocol (Illumina Nextera XT DNA Library Preparation Kit). Each sample was tagged with a unique barcode (96–384 per run), followed by the 2 × 250 bp paired-end sequencing protocol on the Illumina MiSeq instrument. Contigs were constructed from the de-multiplexed MiSeq reads using Geneious software (Version 10.0.9, Biomatters Inc. Newark, NJ). Curated assemblies were validated and annotated by visual inspection and quality control before sequence analysis.

### Subtyping and Genotyping Analysis

The sequences were examined for quality and coverage and assigned an RSV subtype based on the alignment to RSV A and RSV B reference sequences. To make a RSV A and B co-infection call, the ratio of minimum coverage (depth) of the less predominant strain to predominant strain was set at ≥5%. Samples that did not generate at least 1000 mapped reads with at least 4-fold depth of coverage of both F and G genes were marked as “quality/quantity not sufficient” (QNS) and were excluded from the analysis.

Genotypes were assigned based on a best match in a nucleotide BLAST alignment of the second hypervariable region of the G gene against a database of reference sequences with known genotypes^53^. The assigned genotypes were confirmed by inspecting the clustering of sequences observed in a phylogenetic tree built from a joint set of all study sequences and the reference database sequences.

### AA Sequence Analysis of the RSV F and G HVR2

The F gene sequences in FASTA format were translated to AA sequences and aligned to the reference F sequences derived from Netherlands RSVA/13–005275 (GenBank accession no. KX858757) and Netherlands RSVB/13-001273 (GenBank accession no. KX858756). Amino acid variations were determined from pairwise alignments of sample sequences to the references, which were reported as AA variant tables for the RSV A F and RSV B F proteins. Similarly, the G HVR2 sequences were aligned against reference sequences derived from HVR2 of Netherlands RSVA/13-005275 (GenBank accession no. KX858754) and Netherlands RSVB/13-001273 (GenBank accession no. KX858755) to generate AA variant tables. Due to incomplete sequences of some samples from the 2015–16 season, only G HRV2 of season 2 were included in the analysis.

Evolutionary analyses were conducted in MEGA7^54^. RSV F and G gene sequences were translated into protein sequences and aligned using Muscle along with the reference sequences KX858757.1 and KX858756.1 for RSV A F and RSV B F, and KX858754.1 and KX858755.1 for RSV A G and B G, respectively. The evolutionary history was inferred by using the Maximum Likelihood method based on the JTT matrix-based model. Phylogenetic trees were visualized and annotated using ITOL v3^55^. Trees were rooted to their respective reference sequences and annotated with metadata described in figure legends.

F proteins are visualized with PyMOL Molecular Graphics System, Version 2.0 (Schrödinger, LLC). Protein data bank (PDB) files 5UDE^18^ and 3RRR^26^ are used for the pre-fusion F and post-fusion F forms. Amino acid variations in the antigenic sites with frequency >1% are highlighted in black.
